# Antimicrobial resistance profiles of *Escherichia coli* isolated from clinical and environmental samples: findings and implications

**DOI:** 10.1093/jacamr/dlae061

**Published:** 2024-04-27

**Authors:** Maisa Kasanga, Doreen Mainza Shempela, Victor Daka, Mark J Mwikisa, Jay Sikalima, Duncan Chanda, Steward Mudenda

**Affiliations:** Department of Epidemiology and Biostatistics, School of Public Health, Zhengzhou University, Zhengzhou, China; Laboratory Department, Churches Health Association of Zambia, CHAZ COMPLEX Meanwood Drive (off Great East Road), Plot No. 2882/B/5/10, P.O. Box 34511, JC9H+VFF, Lusaka, Zambia; Public Health Department, Michael Chilufya Sata School of Medicine, Copperbelt University, Ndola, Zambia; Department of Pathology and Microbiology, Lusaka Trust Hospital, Plot 2191, H8CC+52F, Nsumbu Rd, Woodlands, Lusaka, Zambia; Laboratory Department, Churches Health Association of Zambia, CHAZ COMPLEX Meanwood Drive (off Great East Road), Plot No. 2882/B/5/10, P.O. Box 34511, JC9H+VFF, Lusaka, Zambia; Adult Centre of Excellence, University Teaching Hospital, Lusaka, Zambia; Department of Pharmacy, School of Health Sciences, University of Zambia, Lusaka, Zambia

## Abstract

**Background:**

The overuse and misuse of antimicrobials has worsened the problem of antimicrobial resistance (AMR) globally. This study investigated the AMR profiles of *Escherichia coli* isolated from clinical and environmental samples in Lusaka, Zambia.

**Methods:**

This was a cross-sectional study conducted from February 2023 to June 2023 using 450 samples. VITEK^®^ 2 Compact was used to identify *E. coli* and perform antimicrobial susceptibility testing. Data analysis was done using WHONET 2022 and SPSS version 25.0.

**Results:**

Of the 450 samples, 66.7% (*n* = 300) were clinical samples, whereas 33.3% (*n* = 150) were environmental samples. Overall, 47.8% (*n* = 215) (37.8% clinical and 10% environmental) tested positive for *E. coli*. Of the 215 *E. coli* isolates, 66.5% were MDR and 42.8% were ESBL-producers. Most isolates were resistant to ampicillin (81.4%), sulfamethoxazole/trimethoprim (70.7%), ciprofloxacin (67.9%), levofloxacin (64.6%), ceftriaxone (62.3%) and cefuroxime (62%). Intriguingly, *E. coli* isolates were highly susceptible to amikacin (100%), imipenem (99.5%), nitrofurantoin (89.3%), ceftolozane/tazobactam (82%) and gentamicin (72.1%).

**Conclusions:**

This study found a high resistance of *E. coli* to some antibiotics that are commonly used in humans. The isolation of MDR and ESBL-producing *E. coli* is a public health concern and requires urgent action. Therefore, there is a need to instigate and strengthen interventional strategies including antimicrobial stewardship programmes to combat AMR in Zambia.

## Introduction

Antibiotics have been a cornerstone in treating infectious diseases, thereby improving patients’ quality of life and life expectancy.^1^ Their discovery marked one of the greatest achievements in the medical field.^2^ However, their overuse and misuse have contributed to the emergence of antimicrobial resistance (AMR), which occurs when microorganisms resist the lethal effects of antimicrobials.^3–8^ AMR is a public health threat affecting humans, animals and the environment; therefore; addressing this problem requires a One Health approach and promotion of antimicrobial stewardship (AMS) programmes.^9–12^ It is suggested that if this problem is not prioritized and addressed, there will be an estimated 10 million human deaths by 2050 and at least 28 million people will live in poverty as a result.^13–16^ Therefore, there is an urgent need for more investments in the fight against AMR using a collaborative ‘One Health’ approach.^17–19^

*Escherichia coli* is a Gram-negative bacterium that causes several infections including urinary tract, respiratory tract, gastrointestinal tract, bloodstream and CNS infections such as meningitis in humans.^20–23^  *E. coli* has developed resistance to many antimicrobials using the natural resistance process.^24^ Additionally, the overuse and misuse of antimicrobials have led to the high level of resistance to these drugs in *E. coli*. This high resistance of *E. coli* to antimicrobials has been reported in human health,^25–28^ animal health^29–33^ and the environment.^25,34^ The occurrence of MDR pathogens in both human and animal health continues to be a public health problem.^35,36^ Consequently, antimicrobial-resistant *E. coli* may cause infections that are difficult or impossible to treat thereby leading to increased morbidity and mortality.^37,38^

Some drivers of AMR in human health include the inappropriate prescribing and dispensing of antimicrobials for the prevention and treatment of various diseases.^39–41^ Reports have shown that most prescribers prescribe antibiotics without microbiological evidence of infection.^42–44^ Additionally, prescribers tend to use their personal experience rather than consulting treatment guidelines when prescribing antibiotics.^43,45^ Consequently, all these practices have been partially attributed to the lack of awareness, limited knowledge, negative attitudes and poor practices of healthcare workers towards AMS.^46–49^ These factors have been worsened due to a lack of diagnostic services in most healthcare facilities, especially in low- and middle-income countries (LMICs).^50,51^ Additionally, self-medication practices and access to antimicrobials without a prescription have increased and are potential drivers of AMR.^40,52–54^ In animal health, farmers overuse and misuse antibiotics for growth promotion, disease prevention, increasing production and therapeutic purposes.^55–58^ These practices expose bacteria to antibiotics and may cause them to develop AMR. Consequently, resistant bacteria can be transmitted from animals to humans. Finally, the use of antimicrobials in the environment and its ecological systems like agriculture and aquaculture have also been linked to the emergence of AMR.^59,60^

Addressing AMR requires a collaborative multidisciplinary One Health approach because AMR affects humans, animals and the environment.^18,19,61–63^ Moreover, addressing AMR requires the actualization of the Global Action Plan on AMR through National Action Plans (NAPs).^64^ Additionally, establishing and implementing AMS programmes in hospitals and communities is essential in combatting AMR.^19,63^ AMS programmes have been reported to reduce inappropriate prescribing, dispensing and use of antibiotics.^63,65–71^ Further, addressing AMR requires establishing and implementing effective surveillance systems, such as those established by the WHO through the Global Antimicrobial Resistance and Use Surveillance System (GLASS).^72^ To meet the objectives of surveillance, laboratories have been crucial in the surveillance of diseases and AMR.^73,74^

In Zambia, there is evidence of antimicrobial-resistant *E. coli* isolated from humans and animals.^25–27,29,31^ This shows that AMR is a challenge in Zambia and requires adequate strategies to address it. The Zambia National Public Health Institute (ZNPHI) through the AMR Coordinating Committee (AMRCC) developed the NAP on AMR, which guides the healthcare authorities on how to tackle AMR.^75,76^ However, there is still a paucity of information on the AMR profiles of *E. coli* isolated from the environment and clinical sources. Based on the above evidence of AMR, this study investigated the AMR profiles of *E. coli* isolated from clinical and environmental samples in Lusaka, Zambia.

## Materials and methods

### Study design and site

**Figure 1.**
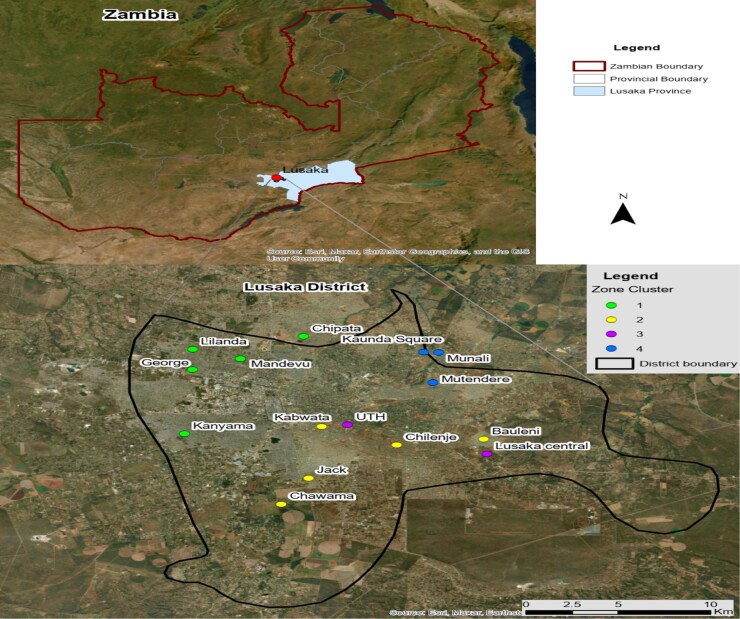
Townships sampled in the Lusaka district.

This was a cross-sectional study conducted between February 2023 and June 2023 in Lusaka city, Zambia. Samples were collected from the University Teaching Hospitals (UTH) including clinical samples (blood, CSF, stool and urine) from both inpatients and outpatients. Environmental samples were collected from both the townships in Lusaka and UTH and included meat, fruits, vegetables, water and hospital equipment. The UTH is the country's largest referral healthcare facility, with a bed capacity of more than 1665, and provides specialist patient care services. Lusaka province has an estimated 687 923 households^77^ and an approximate human population of 3 079 964.^78^ The sampling was done from 14 townships including Chipata Compound, Kaunda Square, Munali, Mandevu, Mtendere, Chilenje, Bauleni, Lusaka Central, Jack Compound, Chawama, Kanyama, Kabwata, Lilanda and George Compound (Figure 1).

### Sample size estimation and sampling

The sample size was estimated using the Cochran formula assuming a prevalence of *E. coli* of 64.5% as determined from a previous study in Zambia.^25^ Assuming an error rate of 5% and a non-response of 20% we determined the minimum sample size required to be 423. The sampling was conducted consecutively using convenience sampling. Only samples stored at room temperature for no more than 2 h were considered for the investigation. Samples that had incomplete demographic information were excluded from the study.

### Data collection

The data collected from clinical samples included the date and time of sample collection, the type of sample collected, an identifying code, and the age and gender of the patients. Environmental samples provided additional information on the source, type and sampled area.

### Quality control

The quality control (QC) of all the culture media used in the study was performed at the media preparation stage and included the culture medium name, brand, colour, gross appearance, texture and quantity of the weighed powdered media. This was followed by measuring the amount of water to be used and the pH of the broth. After media preparation, QC was done for the performance of the media where the physical appearance (e.g. wrinkling and the presence of precipitates), sterility and the use of *E. coli* ATCC 25922 strains for antimicrobial susceptibility testing were done. The internal QC was conducted following the CLSI 2020 guidelines.^79^

### E. coli isolation and identification

All clinical and environmental samples were cultured on MacConkey agar at 37°C for 24 h. The identification of *E. coli* was determined based on its colony morphology, pigment production and Gram-staining reaction followed by confirmation of isolates using biochemical assays. Therefore, *E. coli* was identified when the isolate tested positive for indole and lysine but negative for citrate and urea. Additionally, positive *E. coli* isolates produced a gas, fermented mannitol, and were motile. Further bacterial identification, antibiotic susceptibility testing (AST) and phenotypic detection of ESBL production were conducted using VITEK 2 Compact according to the manufacturer's instructions.

### Identification of E. coli isolates by VITEK 2

We used the VITEK^®^ 2 Compact system (bioMérieux, Marcy-l'Etoile, France) to identify the microorganisms and their susceptibility to antimicrobial agents by examining the biochemical reactions present on microbial identification cards using advanced colorimetry technology. The system's internal optics can read these cards after inoculating them with an unknown organism. Comparing the results to known reactions in the VITEK 2 database enables the system to identify the organism. The system employs a transmittance optical system to interpret the test reactions using different wavelengths within the visible spectrum. During incubation, each reaction is measured every 15 min for turbidity or coloured substrate metabolic products, and an algorithm is employed to remove false readings caused by small bubbles.

The bacterial identification was carried out using an ID-GN card for Gram-negative bacteria (bioMérieux, Marcy-l'Etoile, France). The card is used for automated identification and is designed to identify 135 types of Gram-negative bacilli that are important for fermenting and non-fermenting. To create a bacterial suspension, 3.0 mL of sterile saline was transferred into two clear plastic test tubes, and morphologically similar colonies (pure culture) were added to the first tube using a sterile loop. The density of the suspension with the equivalent standard of 0.50 to 0.63 McFarland was then measured using a turbidity meter (DensiChek), and a 145 μL portion of the suspension was transferred to a second tube. The first vial of bacterial suspension was used for identification, whereas the second was used for AST and ESBL testing.

### Antimicrobial susceptibility testing

AST was done using the AST-GN86 card (bioMérieux SA, France). This test provides an efficient solution for both AST and ESBL tests. A comprehensive susceptibility test for 19 antibiotics from different classes, including penicillins, β-lactamase inhibitor combinations, cephalosporins, carbapenems, aminoglycosides, fluoroquinolones, tetracycline, nitrofurantoin and trimethoprim/sulfamethoxazole, was conducted using the AST-GN86 card in conjunction with the VITEK 2 system. The results of the susceptibility test were interpreted as sensitive, intermediate or resistant according to the CLSI 2020 guidelines.^79^ Furthermore, the VITEK 2 system determines the MIC for ultimate accuracy. MDR is defined as non-susceptibility to at least one agent in three or more antimicrobial categories. A confirmatory test called the VITEK 2 ESBL test is used to find out whether *E. coli* AST-GN86 cards contain ESBLs. In this study, MDR was defined as non-susceptibility to at least one agent in three or more antibiotic classes; XDR was defined as non-susceptibility to at least one agent in all but two or fewer antimicrobial categories, i.e. bacterial isolates remain susceptible to only one or two antimicrobial categories; Pandrug resistance (PDR) was defined as non-susceptibility to all antibiotics tested.^80^

### Data analysis

The raw data from the VITEK^®^ 2 Compact system were entered in Microsoft Excel, cleaned, coded and analysed using Statistical Package for Social Sciences (SPSS) version 25.0 (IBM Corp., Armonk, NY, USA). We used frequencies to describe the distribution of sociodemographic, clinical and environmental variables, and resistance profiles of *E. coli* to a panel of antibiotics. The prevalence of MDR and ESBL-producing *E. coli* was determined using descriptive statistics. A *P* < 0.05 was regarded as statistically significant.

## Results

A total of 450 samples, (66.7%, *n* = 300 clinical samples; 33.3%, *n* = 150 environmental samples) were collected and cultured. The demographic characteristics of age and gender were only applicable to specimens from patients, which included 176 females (58.7%) and 124 males (41.3%); the median age was 30 years. Most samples were collected from patients aged 25–34 years (22.0%), 0–14 years (21%), and those aged 55 years and above (20.3%) (Table 1).

**Table 1.** Sociodemographic characteristics of patients

Variables	Frequency (*n*)	Percentage
Age,^a^ y		
0–14	63	21.0
15–24	50	16.7
25–34	66	22.0
35–44	30	10.0
45–54	30	10.0
55 and above	61	20.3
Gender		
Female	176	58.7
Male	124	41.3

^a^Age summary: median age = 30 years; IQR = 30 years.

### Sources and isolation rates of E. coli isolates

Overall, 215 (47.8%) *E. coli* isolates were isolated from the 450 samples, of which 170/450 (37.8%) were from clinical and 45/450 (10%) from environmental sources. Environmental samples included fruits and vegetables (*n* = 11; 2.4%), water (*n* = 12; 2.7%), hospital equipment (*n* = 18; 4%) and meat (*n* = 4; 0.9%), whereas clinical samples included blood (*n* = 6; 1.33%), CSF (*n* = 6; 1.333%), pus (*n* = 29; 6.44%), stool (*n* = 1; 0.22%) and urine (*n* = 128; 28.44%). Most of the environmental samples were from hospital equipment and water, whereas most of the clinical samples were from urine and pus (Table 2).

**Table 2.** Descriptive characteristics of the source of specimens (*n* = 450)

Variables	Frequency (*n*)	Percentage
Negative environmental samples	105	23.3
Positive environmental samples	45	10.0
Fruits and vegetables	11	2.4
Water	12	2.7
Hospital equipment	18	4.0
Meat/other foods	4	0.9
Negative clinical samples	130	28.9
Positive clinical samples	170	37.8
Blood	6	1.33
CSF	6	1.33
Pus	29	6.44
Stool	1	0.22
Urine	128	28.44
Wards (*n* = 170)		
Admission	6	3.5
Casualty	2	1.2
ICU^a^	13	7.7
Medical	17	10.0
Obs and Gynae	10	5.9
Paediatrics	17	10.0
Surgery	39	22.9
Outpatient	66	38.8
Total		100

Obs and Gynae, Obstetrics and Gynaecology unit.

^a^The ICU is comprised of a neonatal unit, paediatrics unit and adult unit.

### AST patterns of all 215 E. coli isolates

From the 215 *E. coli* isolates, most were resistant to ampicillin (81.4%), sulfamethoxazole/trimethoprim (70.7%), ciprofloxacin (67.9%), levofloxacin (64.6%), cephazolin (62.8%), ceftriaxone (62.3%), cefuroxime (62%) and cefixime (51.2%). However, some *E. coli* isolates were highly susceptible to amikacin (100%), meropenem (99.5%), imipenem (99%), ertapenem (98.6%), nitrofurantoin (89.3%), ceftolozane/tazobactam (82%), gentamicin (72.1%) and piperacillin/tazobactam (68.8%) (Table 3).

**Table 3.** Antibiotic susceptibility patterns of *E. coli* isolates

Antibiotics	Antibiotic susceptibility patterns of all *E. coli* isolates (*n* = 215)		
	Susceptible	Intermediate	Resistant
AMK	215 (100%)	0 (0%)	0 (0%)
GEN	155 (72.1%)	14 (6.5%)	46 (21.4%)
ETP	212 (98.6%)	2 (0.9%)	1 (0.5%)
IPM	213 (99%)	1 (0.5%)	1 (0.5%)
MEM	214 (99.5%)	0 (0%)	1 (0.5%)
CFZ	62 (28.8%)	18 (8.4%)	135 (62.8%)
CXM	78 (36%)	4 (2%)	133 (62%)
CAZ	105 (48.8%)	12 (5.6%)	98 (45.6%)
CRO	81 (37.7%)	0 (0%)	134 (62.3%)
FEP	105 (48.8%)	0 (0%)	110 (51.2%)
C/T	176 (82%)	0 (0%)	39 (18%)
AMP	40 (18.6%)	0 (0%)	175 (81.4%)
AMC	77 (35.8%)	59 (27.4%)	79 (36.7%)
TZP	148 (68.8%)	14 (6.5%)	53 (24.7%)
CST	0 (0%)	213 (99%)	2 (1%)
SXT	63 (29.3%)	0 (0%)	152 (70.7%)
NIT	192 (89.3%)	14 (6.5%)	9 (4.2%)
CIP	68 (31.6%)	1 (0.5%)	146 (67.9%)
LVX	75 (34.9%)	1 (0.5%)	139 (64.6%)

AMC, amoxicillin/clavulanate; AMK, amikacin; AMP, ampicillin; CAZ, ceftazidime; CFZ, cefazolin; CIP, ciprofloxacin; CRO, ceftriaxone; CST, colistin; C/T, ceftolozane/tazobactam; CXM, cefuroxime; ETP, ertapenem; FEP, cefepime; GEN, gentamicin; IPM, imipenem; LVX, levofloxacin; MEM, meropenem; NIT, nitrofurantoin; SXT, sulfamethoxazole/trimethoprim; TZP, piperacillin/tazobactam.

### Prevalence of MDR and ESBL-producing E. coli

**Figure 2.**
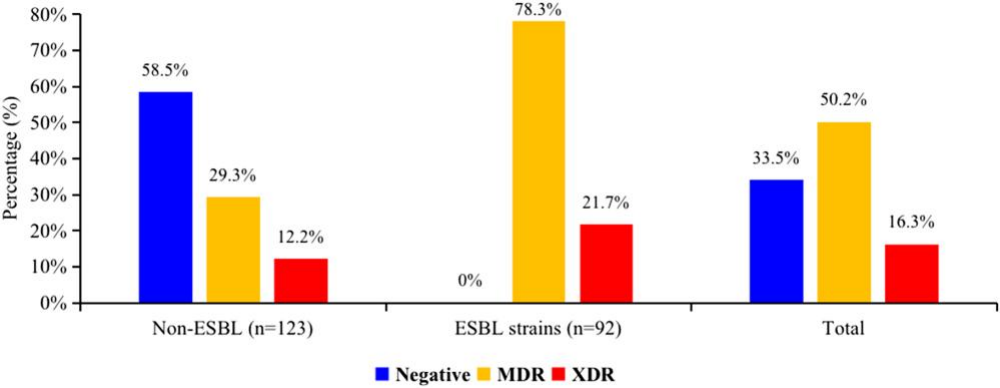
Distribution of MDR and ESBL-producing *Escherichia coli*.

Overall, of 215 *E. coli* isolates, 92 (42.8%) were ESBL producers, of which 74/92 (80.4%) were from clinical samples and 18/92 (19.6%) were from environmental samples. Of the 215 *E. coli* isolates, 143 (66.5%) were MDR. Additionally, 72 (78.3%) of the ESBLs were MDR and 20 (21.7%) were potential XDR (Figure 2).

## Discussion

This study investigated the AMR profiles of *E. coli* isolated from clinical and environmental samples in selected sites of the Lusaka district in Zambia. The prevalence of *E. coli* was found to be 47.8%, of which 37.8% were clinical isolates and 10% were environmental isolates. Most of the *E. coli* isolates were resistant to ampicillin (81.4%), sulfamethoxazole/trimethoprim (70.7%), ciprofloxacin (67.9%), levofloxacin (64.6%), cephazolin (62.8%), ceftriaxone (62.3%), cefuroxime (62%) and cefixime (51.2%). However, the *E. coli* isolates were highly susceptible to amikacin (100%), meropenem (99.5%), imipenem (99%), ertapenem (98.6%), nitrofurantoin (89.3%), ceftolozane/tazobactam (82%), gentamicin (72.1%) and piperacillin/tazobactam (68.8%). A total of 42.8% of the *E. coli* isolates were ESBL-producers and 66.5% were MDR, whereas 78.3% of the ESBL producers were MDR, of which 21.7% were potential XDR.

Our study found that the prevalence of *E. coli* isolates was 47.8% (37.8% clinical and 10.0% environmental). The prevalence of *E. coli* clinical isolates in our study was lower than the 58.9% reported in the Democratic Republic of Congo (DRC).^81^ Further, the prevalence of *E. coli* isolates from environmental sources in our study was lower than that found from the watershed and food-producing animals in the USA (99.3%)^82^ and in China (64.7%).^83^ Also, our study found a lower prevalence of *E. coli* isolated from environmental sources compared with those reported in Nigeria and Ghana, where the *E. coli* prevalence was 48.9% from raw milk and 49.5% from drinking water sources.^84,85^ We reported a higher prevalence of *E. coli* clinical isolates compared with the 30.3% reported in Palestine^86^ and 14.2% in Ethiopia.^87^ These variations could be partly due to geographical variations, technical differences, the methods used and the experience of the personnel who conducted the laboratory work.

Our study found that *E. coli* was highly resistant to ampicillin, which is consistent with other studies, indicating that penicillins are widely used in clinical and environmental settings.^88–92^ Penicillins are widely accessed without prescription and are usually inappropriately used, which might contribute to the observed resistance to these drugs.^93^ The resistance of *E. coli* to penicillins could also be facilitated by the presence of AmpC β-lactamases encoded by the chromosome of *E. coli*.^94^ Additionally, our study revealed that the *E. coli* isolates were highly resistant to sulfamethoxazole/trimethoprim. Our findings corroborate reports from other studies in which *E. coli* were found to be highly resistant to sulfamethoxazole/trimethoprim.^92,95–97^ The overuse and misuse of sulfamethoxazole/trimethoprim have contributed to the resistance of *E. coli* to this drug combination. However, the resistance of *E. coli* to sulfamethoxazole/trimethoprim has been reported even in individuals who have never used the drug combination.^98^

Our study also revealed high resistance of *E. coli* to ciprofloxacin and levofloxacin. These findings corroborate reports from other studies that found that *E. coli* had developed high resistance to ciprofloxacin and levofloxacin.^90,99–102^ These resistance patterns reported in our study and comparable studies could be due to the misuse of quinolones for the treatment of urinary tract infections and respiratory tract infections.^103–106^ The resistance of *E. coli* to quinolone antibiotics could be due to the occurrence of chromosomal mutations or plasmid-mediated quinolone resistance.^107^ Furthermore, *E. coli* may also harbour chromosomally encoded AmpC β-lactamases that are capable of hydrolysing cephalosporins, especially when overexpressed.^94^ Unfortunately, even after a reduction in prescriptions of fluoroquinolones like ciprofloxacin, there appears to be an increase in ciprofloxacin-resistant *E. coli*.^101^

The current study also found that most *E. coli* isolates were resistant to cephalosporins, including cephazolin, ceftriaxone, cefuroxime and cefepime. In Zambia, increased resistance of *E. coli* clinical isolates to ceftriaxone was reported in previous studies and was revealed to be due to high use of this drug in healthcare facilities.^108,109^ Two studies conducted in Iraq reported high resistance of *E. coli* to ceftriaxone and cefotaxime.^89,90^ A study in the DRC found high resistance of *E. coli* to ceftazidime.^110^ Consequently, the resistance of *E. coli* to antibiotics could be due to the presence of ESBLs that are capable of hydrolysing these drugs, especially the first-, second- and third-generation cephalosporins.^111–113^ Hence, it is critical to conduct molecular surveillance of *E. coli* isolated from clinical and environmental samples.^114–117^

The present study found that *E. coli* was highly susceptible to amikacin, meropenem, imipenem, ertapenem, nitrofurantoin, gentamicin and ceftolozane/tazobactam. Our findings are in line with those reported in other studies where *E. coli* was 90%–100% susceptible to amikacin.^118^ Additionally, the high susceptibility of *E. coli* isolates to carbapenems has been reported in other studies.^89,119^ Intriguingly, the high susceptibility of *E. coli* to nitrofurantoin and gentamicin has also been reported by other researchers.^89^ A 100% susceptibility of *E. coli* to nitrofurantoin was reported in another study.^95^ These findings are encouraging because nitrofurantoin is used in many countries as a first-line and empirical treatment for urinary tract infections.^95^

A high prevalence of MDR *E. coli* (66.5%) was reported in the current study. A comparable but slightly lower prevalence (64.9%) of MDR *E. coli* was reported in Nepal,^120^ with a lower rate (48.7%) reported in Ghana.^121^ However, a higher prevalence of MDR *E. coli* was previously reported in Sudan (92.2%),^122^ Zimbabwe (84%)^92^ and Nigeria (79.2%).^123^ MDR *E. coli* is a public health concern and has been associated with increased morbidity and mortality globally.^124^ This is because MDR infections limit the choice of antimicrobial therapy and make the treatment of infections difficult or impossible.^125,126^ The high prevalence of MDR *E. coli* reported in our study and similar studies indicates the extent of this problem globally. Addressing this problem requires a multifaceted holistic approach.

The present study found that 42.8% of *E. coli* were ESBL-producing. Their occurrence in this study indicates the high resistance to antibiotics including penicillins, third-generation cephalosporins (ceftriaxone, cefotaxime and ceftazidime) and aztreonam, similar to reports from other studies.^127,128^ This is because ESBLs hydrolyse these antibiotics leading to treatment failure.^111,112^ Comparable to our findings, a Chinese study reported that 45.7% of *E. coli* isolated from blood and respiratory samples were ESBL-producing.^118^ Lower prevalences of ESBL-producing *E. coli* were reported in Nigeria (16.7%)^123^ and in Nepal (38.9%),^120^ whereas higher rates were found in previous studies, including 59% in the Central African Republic^129^ and 91% in the USA.^130^ The presence of ESBL-producers is of clinical significance and if not addressed may complicate treatment leading to treatment failure and eventually increased mortality.^131,132^

Our study highlights the high AMR of *E. coli* to some of the antibiotics commonly used in humans and the environment. Therefore, the study findings demonstrate the need for heightened surveillance of AMR in humans and the environment. Additionally, our findings demonstrate the need for heightened AMS programmes in hospitals and communities.

### Conclusions

This study found that *E. coli* was highly resistant to some common antibiotics used in humans and the environment. Interestingly, some isolates were highly susceptible to some priority antibiotics used in humans. However, the high prevalence of MDR and ESBL-producing *E. coli* in this study is of public health concern and can affect the treatment of infections. There is a need to heighten the surveillance of AMR in humans and the environment. Further, AMS programmes should be instigated and strengthened in healthcare facilities and in communities to reduce the emergence of AMR and prevent its potentially fatal consequences.
